# Evaluating an Alleged Mimic of the Monarch Butterfly: *Neophasia* (Lepidoptera: Pieridae) Butterflies are Palatable to Avian Predators

**DOI:** 10.3390/insects9040150

**Published:** 2018-10-29

**Authors:** Dale A. Halbritter, Johnalyn M. Gordon, Kandy L. Keacher, Michael L. Avery, Jaret C. Daniels

**Affiliations:** 1 USDA-ARS Invasive Plant Research Laboratory, 3225 College Ave, Fort Lauderdale, FL 33314, USA; 2 Entomology and Nematology Department, University of Florida, 1881 Natural Area Dr, Steinmetz Hall, Gainesville, FL 32611, USA; jdaniels@flmnh.ufl.edu; 3 Fort Lauderdale Research and Education Center, University of Florida, 3205 College Ave, Davie, FL 33314, USA; johnalynmgordon@ufl.edu; 4 Florida Field Station, USDA-APHIS National Wildlife Research Center, 2820 E University Ave, Gainesville, FL 32641, USA; kandy.l.keacher@aphis.usda.gov (K.L.K.); gibs8387@gmail.com (M.L.A.); 5 2906 NW 14th Pl., Gainesville, FL 32605, USA; 6 McGuire Center for Lepidoptera and Biodiversity, Florida Museum of Natural History, 3215 Hull Road, Gainesville, FL 32611, USA

**Keywords:** bird, butterfly, *Danaus plexippus*, mimicry, *Neophasia*, palatability, predation

## Abstract

Some taxa have adopted the strategy of mimicry to protect themselves from predation. Butterflies are some of the best representatives used to study mimicry, with the monarch butterfly, *Danaus plexippus* (Lepidoptera: Nymphalidae) a well-known model. We are the first to empirically investigate a proposed mimic of the monarch butterfly: *Neophasia terlooii*, the Mexican pine white butterfly (Lepidoptera: Pieridae). We used captive birds to assess the palatability of *N. terlooii* and its sister species, *N. menapia*, to determine the mimicry category that would best fit this system. The birds readily consumed both species of *Neophasia* and a palatable control species but refused to eat unpalatable butterflies such as *D. plexippus* and *Heliconius charithonia* (Lepidoptera: Nymphalidae). Given some evidence for mild unpalatability of *Neophasia*, we discuss the results considering modifications to classic mimicry theory, i.e., a palatability-based continuum between Batesian and Müllerian mimicry, with a quasi-Batesian intermediate. Understanding the ecology of *Neophasia* in light of contemporary and historical sympatry with *D. plexippus* could shed light on the biogeography of, evolution of, and predation pressure on the monarch butterfly, whose migration event has become a conservation priority.

## 1. Introduction

Mimicry within animals is evident when one species to some degree matches another in visual appearance, chemical profile, and/or behavior. Butterflies have served as excellent models to investigate mechanisms of mimicry, with emphasis on the Neotropical genus *Heliconius* (Lepidoptera: Nymphalidae) [1,2,3,4,5,6,7], sex-limited mimicry within *Papilio* spp. (Lepidoptera: Papilionidae) [8,9,10,11], and the well-known *Limenitis-Danaus* (Lepidoptera: Nymphalidae) system [12,13,14,15]. Mimics can evade predation because they emulate a toxic, distasteful, or dangerous model that predators learn to identify and avoid. In Batesian mimicry, a mimic emulates another toxic or distasteful species, but the mimic is not toxic or distasteful. The Batesian mimic is considered deleterious to its model, especially if the mimic is comparably abundant [16,17,18,19]. In Müllerian mimicry, a toxic or distasteful species visually mimics another toxic or distasteful species. Müllerian mimicry complexes experience less predation pressure because it is easier for predators to learn to avoid one common pattern. Mimicry may not be confined to a strict dichotomy, as the level of unpalatability may vary in some prey species and different predator species may vary in their perception of unpalatability, adding complexity to the spectrum between Batesian and Müllerian mimicry [20]. However, there is often a fine threshold within the palatability spectrum at which predators reject their prey [17], suggesting that classical Batesian and Müllerian mimicry would be most common [19]. Additionally, the spectrum is likely driven by variations in the reactions of different predator species when they encounter the mimic [17].

**Figure 1:**
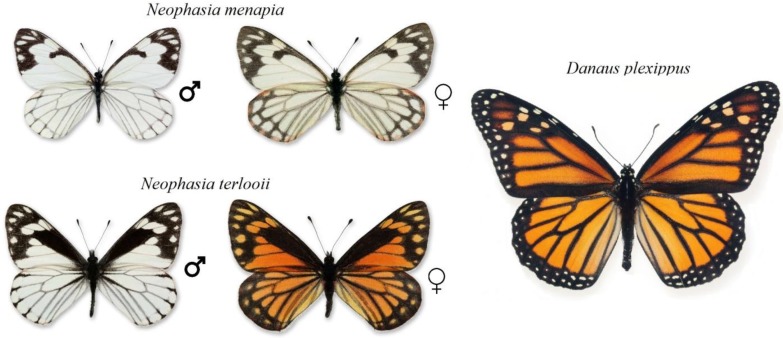
A visual comparison between allegedly mimetic *Neophasia* spp. (Lepidoptera: Pieridae) and their proposed model, *Danaus plexippus* (Lepidoptera: Nymphalidae). Based on the visible spectrum, female *N. terlooii* are the most likely mimics of *D. plexippus*. Photographs by author Dale A. Halbritter.

Our study is the first to empirically examine the nature of an alleged case of mimicry in *Neophasia* (Lepidoptera: Pieridae), an unusual genus of North American butterflies. Female *Neophasia terlooii*, the Mexican pine white butterfly, are orange with black markings resembling the monarch butterfly, *Danaus plexippus* (Figure 1). The suggestion that female *N. terlooii* mimic *D. plexippus* was first published by Poulton [21] and has yet to be empirically investigated. The black markings on male *N. terlooii* resemble those on female *N. terlooii*, but the background color is white in males (Figure 1). Currently, the only other recognized species of *Neophasia* is *N. menapia*, the pine white butterfly, and both males and females are white with black markings, but their black markings are like those on *N. terlooii* (Figure 1). *Neophasia menapia* has one summer brood and is found from southwestern British Columbia to Guadalupe Mountains National Park in extreme southwest Texas [22,23]. *Neophasia terlooii* has a small summer brood and a larger fall brood and is found from the sky islands of southeastern Arizona to central México [22,24]. As larvae, *Neophasia* spp. feed on conifers and we considered the possibility that larvae sequester unpalatable host plant compounds, retaining them through the adult stage and potentially rendering the adults unpalatable to predators.

Under the assumption that *Neophasia* spp. mimic *D. plexippus*, our objective was to determine where *Neophasia* spp. reside on the Batesian-Müllerian mimicry spectrum. In a manner like that employed by Long et al. [25], we used caged birds as representatives of visual predators to determine the butterflies’ palatability. Jones [26,27] was the first to use wild birds to assess palatability and aposematism in insects, and Brower [8,12,13] was the first to empirically investigate mimicry mechanisms in North American butterflies using caged birds. In the latter studies, birds learned to recognize and avoid consuming toxic butterflies and subsequently avoid butterflies that resembled toxic ones. We discuss the implications of *Neophasia* spp., especially female *N. terlooii*, as being closer to Batesian mimics, and, in a greater ecological context, discuss how predation pressure can influence geographic range.

## 2. Materials and Methods

### 2.1. Butterfly Collection

Adult *N. menapia* were collected in July of 2013 and adult *N. terlooii* in October of 2013. To account for potential geographic variations in palatability, *N. menapia* were collected from three sites in northern Arizona (Kaibab Plateau, Mogollon Rim, and White Mountains) separated by at least 150 km and *N. terlooii* were collected from two different mountain ranges in southeastern Arizona (Huachuca and Santa Rita Mountains). *Eurema daira* (Lepidoptera: Pieridae), a smaller, yellow and black palatable species (author Jaret C. Daniels, unpublished data), was collected in Gainesville, Florida in August and September of 2013. Field-collected butterflies were placed into glassine envelopes and later frozen at −15 °C. Envelopes were stored in sealed plastic bags to minimize specimen dehydration and oxidation due to air exposure at subfreezing temperatures. Arizona specimens were first frozen then shipped overnight on ice to the University of Florida.

### 2.2. Aviary Setup

Fish crows (*Corvus ossifragus*) held at the USDA National Wildlife Research Center in Gainesville, Florida were utilized to approximate the palatability of *Neophasia* spp. to generalist avian predators (IACUC #201308135). Although the crows were wild-caught, they had been in captivity for at least 5 y, which is enough time for wild-caught birds to be considered naïve [28], i.e., past encounters with similarly-patterned, distasteful prey would be forgotten and reactions to *Neophasia* would not be confounded. Six crows were housed individually in 1.8 × 1.2 × 1.2 m wire cages in an aviary. The aviary was 14.6 × 12.2 m, had a hard roof, and screened sides to permit airflow. Two to three bare tree branches were placed in each cage for perching, water was offered in dishes on the cage floors, and standard diet and experimental insects were offered on 75 × 75 cm artificial turf mats placed on the cage floors. The standard diet was dog food (Ol’ Roy High Performance), but crows received occasional treats (e.g., raisins and Cheerios cereal). To prevent the birds from seeing each other, white sheets were affixed to three sides of the cages and the fronts were exposed for viewing. Cages were suspended approximately 1 m above the aviary floor.

### 2.3. Presentation of Experimental Insects

Butterflies were thawed, and each bird received one butterfly in a clear, 10 cm diameter plastic dish. To entice the birds to taste the butterflies, one live mealworm (*Tenebrio molitor* Coleoptera: Tenebrionidae) and one thawed adult house cricket (*Acheta domestica* Orthoptera: Gryllidae) were included in each dish. Standard diets were removed by 4 p.m. the afternoon before experiments, the latter of which occurred between 9 a.m. and 11 a.m. the next morning. Standard diets were returned after each assay. Experiments occurred daily during weekdays. As the palatable negative control, *E. daira* were offered initially for several days, followed by the *Neophasia* spp. for the remaining days. *Neophasia menapia* (33 male and 14 female butterflies offered) palatability experiments occurred from August 2013 to early September 2013, and those with *N. terlooii* (30 male and 15 female butterflies offered) occurred from November 2013 to mid-December 2013. Upon completion of the *N. terlooii* experiments in December, *D. plexippus* and *Heliconius charithonia* were offered as unpalatable positive controls. The positive control species were collected in Gainesville, FL in the fall. Birds were individually observed in a random order each day.

### 2.4. Behavioral Observations

A digital video camera mounted on a tripod was used to record bird behavior. For each observation, the experimenter retreated out of the bird’s sight and observed the bird’s silhouette through the sheet from roughly 10 m outside the aviary. Behavior was recorded for 5 min following the bird’s last visit to the cage floor. Head shaking, feather ruffling, and bill wiping are regarded as mild indicators of unpalatability, while violent or repeated head shaking, retching, and vomiting are regarded acute indicators of unpalatability [29]. We ranked each behavior numerically: bill wiping and/or feather ruffling (1), head shaking (2), violent head shaking (3), retching (4), and vomiting (5). Behaviors observed were summed for each bird on each trial day. We checked the dishes, mats, and floor beneath cages for leftover insects after each experiment. Bowls were removed shortly after insects were consumed and standard diets were returned to each cage after the experiment.

### 2.5. Statistical Analyses

Generalized linear models fit with binomial distributions were used to model the effects of bird individual, butterfly species, and the interaction between the two on the proportion of butterflies consumed. A Wald chi-squared test was used to test the significance of the explanatory variables (*p* < 0.05). Only birds that consumed butterflies were included in the analyses of reactions. The summer experiment with the negative control and *N. menapia* was analyzed separately from the fall experiment with the negative control and *N. terlooii*. Wilcoxon signed-rank tests were used to test whether butterfly species or sex (factors with two levels) impacted bird reaction. A Kruskal-Wallis test was used to test whether bird individual (factor with five levels) impacted bird reaction. Additionally, Wilcoxon signed-rank tests were used to compare the reactions of birds that ate only incentive insects to those that ate *Neophasia* spp. and the incentive insects. Data were organized in Microsoft Excel and analyzed using R version 3.4.4 in the RStudio version 1.1.447 development environment [30]. We used the following R packages for analyses: xlsx [31] for importing Excel spreadsheets into R, stats [30] for the generalized linear modeling, Wilcoxon signed-rank and Kruskal-Wallis tests, and aod [32] for the Wald chi-squared test.

## 3. Results

**Figure 2:**
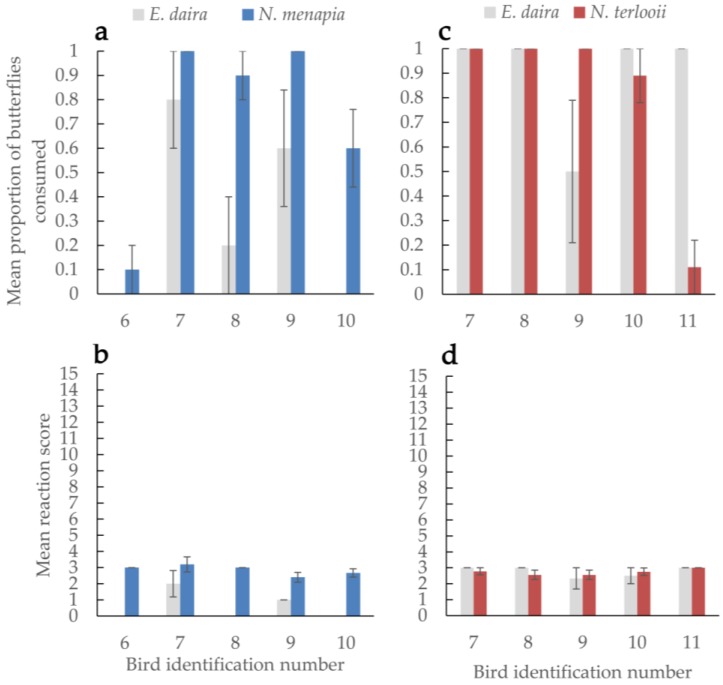
The average proportion of butterflies consumed (**a**,**c**) by each bird and each bird’s average reaction score (**b**,**d**) are compared between a palatable control, *Eurema daira*, and either *Neophasia menapia* (**a**,**b**) or *N. terlooii* (**c**,**d**). Reaction scores range from 0 (no discomfort after consuming a food item) to 15 (all possible symptoms, cumulative from mild to severe, of consuming a food item of uncertain palatability). Brackets indicate standard error of the mean.

Consumption rates of *Neophasia menapia* did not differ from those of *E. daira* (Figure 2a). Overall butterfly consumption rates did not differ between birds, nor was there sufficient evidence to say some birds consumed more of one species of butterfly over another. Of the birds that consumed either *N. menapia* or *E. daira*, consuming *N. menapia* led to a stronger reaction (Figure 2b) (W = 24.5; *p* = 0.036). Bird reactions to consuming male (mean = 2.8 ± 0.115 SEM) versus female (mean = 3.0 ± 0.436 SEM) *N. menapia* did not differ significantly.

Consumption rates of *Neophasia terlooii* did not differ from those of *E. daira* (Figure 2c). Overall butterfly consumption rates did not differ between birds, nor was there sufficient evidence to say some birds consumed more of one species of butterfly over another. Bird reactions to consuming either *N. terlooii* or *E. daira* did not differ*.* Overall, bird reactions to eating any species of butterfly did not differ (Figure 2d). Bird reactions to consuming male (mean = 2.7 ± 0.155 SEM) versus female (mean = 2.7 ± 0.225 SEM) *N. terlooii* did not differ significantly.

Nearly all birds performed a head shake, feather ruffle, and/or bill wipe after consuming butterflies. Birds 6 and 11 rarely consumed butterflies, but they frequently consumed the incentive insects and still performed a head shake, feather ruffle, and/or bill wipe. Feather ruffling was observed in all birds before and after consuming any insect food items. Reactions did not differ between instances when birds ate *Neophasia* spp. and incentive insects and instances when birds only ate incentive insects. Neither the *H. charithonia* nor *D. plexippus* positive controls were consumed by the birds. Birds picked up these unpalatable butterflies and manipulated them in their bills for several minutes before dropping them.

## 4. Discussion

Skinner [33] wrote of *N. terlooii* stating that “the female of the species, was once sent to me as a ‘little *Danais*’ and it really looks like one-Here would be a good opportunity to build up a mimicry theory’’ (Note: “*Danais*” = *Danaus*). Our study is the first to empirically explore this mimicry hypothesis, which was formally proposed by Poulton [21]. *Neophasia* spp. display ecological characteristics that would suggest their unpalatabililty, such as their vibrant and conspicuous coloration and the presence of many distasteful compounds in the larval diet. Weak flight capacity in these species suggests that unpalatability may be used as a defense mechanism by members of this genus. Here, we show that *Neophasia* are more likely Batesian mimics of *D. plexippus*, as they are more palatable to a visual, avian predator than the model. Despite lacking similar orange wing color to the model, the black venation of *N. menapia* is visually similar to the wing venation of *N. terlooii* and ultraviolet reflectance of wings across *Neophasia* spp. may be comparable regardless of background color. Passerine birds, which include crows, are known to see in both the visible and ultraviolet spectra [34] and both attributes should be measured in butterflies in future studies to assess whether it is only female *N. terlooii* that are mimetic.

### 4.1. Batesian vs. Müllerian Mimicry

Mimicry does not always appear to be a dichotomy between Batesian and Müllerian; there can be a continuum within which there are semi-palatable mimics and cases of models and mimics switching roles depending on their respective larval diets and resulting adult palatability [35]. In our case, *Neophasia* is more likely a purely Batesian mimic. Consumption of *N. menapia* elicited a somewhat stronger reaction (i.e., discomfort associated with some degree of unpalatability) than consumption of palatable *E. daira* (this was likely due to one instance when a bird retched after consuming a *N. menapia*, possibly due to a wing getting stuck), but the reaction to *E. daira* was the same as that to *N. terlooii* in the fall. Additionally, the reactions to consuming *N. menapia* and *N. terlooii* were quite similar (Figure 2b,d). Dietary preferences of the crows may have changed in the fall resulting in a greater incentive to consume the smaller *E. daira*. There were no instances of a crow regurgitating after eating *Neophasia* or *Eurema*. Most *Neophasia* were consumed within seconds, unlike the unpalatable butterflies, which were all manipulated by the birds for several minutes until they were subsequently discarded. Incentive insects, *E. daira*, and *Neophasia* spp., despite any variations in palatability, were likely below the threshold of predator rejection based on palatability (see [17]), while *D. plexippus* and *H. charithonia* were above the threshold and therefore rejected. Brief head shakes, feather ruffles, and bill wipes seemed to be fairly common behaviors after consuming *Neophasia*, which may have been associated with subtle unpalatability. However, crickets and mealworms are palatable, and these behaviors were still observed after crows consumed these insects, which suggests these behaviors may be more baseline or there may have been other stressors causing these subtle reactions.

### 4.2. Predator‒Prey Dynamics

Fish crows, although not sympatric with either *Neophasia* spp., make suitable surrogates for avian predators in *Neophasia* habitat. Their congeners, American crows, *Corvus brachyrhynchos*, co-occur with *Neophasia* and invertebrates are a part of their diet [36]. Additionally, other members of Corvidae, such as jays, will prey on invertebrates [36]. Most importantly, taste receptors are fairly conserved at the order level within birds. Passerines (which includes Corvidae) have between eight and ten Tas2r genes that enable the detection of bitterness and thereby likely toxic compounds [37]. Therefore, it is plausible that passerine birds in *Neophasia* habitat would exhibit a behavioral response comparable to the fish crows our study.

Predation pressure can shape species ranges and this may impact the southern range limit of *N. menapia*, which, based on its color, is a less likely mimic of *D. plexippus*. In considering dispersal barriers and environmental gradients, coevolved species interactions have potential to limit species distributions [38]. *Neophasia terlooii* is thought to have evolved in montane central México [39], a region that is periodically saturated with congregating monarch butterflies during their annual migration. The high abundance of a toxic model may have then resulted in selection favoring mimetic *Neophasia*. To our knowledge, there are no records of *N. terlooii* co-occurring with monarchs at their overwintering sites in México. As the distribution of *N. terlooii* moved north, co-occurrence with monarchs became restricted to migrating individuals. Because monarchs do not occur in as great a density as *N. menapia*, nor are they migrating when *N. menapia* are flying, developing an orange color may not have been as advantageous. Visual predators in the mountains of central México would likely be more experienced in recognizing and avoiding monarchs, and thereby avoid butterflies with convincing similarities in appearance. Predation pressure within the mountains of northern and central México could play a role in limiting the southern range boundary of *N. menapia*. Additional research is needed to determine the abundance and species composition of potential avian predators within the ranges of both *Neophasia* spp.

*Neophasia menapia* is known to have periodic population irruptions [40,41], situations in which unpalatable models could be overwhelmed by a palatable mimic. Smith et al. [42] postulated that *Danaus chrysippus* can escape from an overabundance of palatable *Hypolimnas* spp. (Lepidoptera: Nymphalidae) mimics via polymorphism. However, models are typically under strong purifying selection, with rare variants being mistakenly preyed upon and therefore selected against [20]. To our knowledge, there are no documented cases of *N. terlooii* population irruptions in the mountains of northern México, but it certainly warrants monitoring given the similarity in life history it shares with *N. menapia* and the impacts climate change can have on insect population dynamics [43]. Population irruptions of *N. terlooii* that occur prior to the arrival of overwintering monarchs could impact predation rates on the monarchs. As stated earlier, an overabundance of a palatable mimic can negatively impact the unpalatable model because the predators’ negative association with the shared wing pattern diminishes and the models suffer from increased predation [16,17,18,19]. We found the sex ratios in *Neophasia* to be highly male-skewed. If mimicry is limited to females, the abundance of the mimic and deleterious effect on the model may be less than it appears.

### 4.3. Sex-Limited Mimicry

In Batesian mimicry complexes, female butterflies are often mimetic and males non-mimetic [44], a subtype of Batesian mimicry known as female-limited polymorphic mimicry (FPM) [25,45,46]. In [44], birds tended to prefer female butterflies because they are easier to capture and more nutritious. In cases where species exhibited mimetic and non-mimetic female forms, the non-mimetic forms were attacked with greater frequency, while mimetic forms and males were attacked less frequently. Evolutionarily, the phenologies of the model and mimic are important to understand. Long [46] found strong evidence in favor of the model-first hypothesis [47,48], in which the model and mimic benefit when the model emerges first. In this situation, predators can learn to avoid the model and mimics that emerge later. However, in the FPM subtype, the emergence of the mimic can occur before the model [46]. Male *N. terlooii* were observed in early October while females did not increase in abundance until late October (author Dale A. Halbritter, unpublished data). Early to mid-October is roughly the peak of the monarch migration through southern Arizona [49]. Therefore, in contrast to males, the mimetic females would be more likely to emerge after the model is present and after predators have learned to avoid the pattern on the model.

## 5. Conclusions

Future directions of this research to determine whether *Neophasia* spp. are truly mimetic, would be to perform experiments with naïve birds in a manner like that employed by Jones [26,27], Brower [8,12,13], and Long et al. [25]. If birds refuse to consume *N. terlooii* females after learning to avoid monarchs, there will be strong evidence for a mimetic relationship. If the same occurs with male *N. terlooii* and both sexes of *N. menapia*, mimicry in the ultraviolet spectrum should be considered. This would provide more evidence for mimicry in a visual spectrum that is overlooked. Nonetheless, we provide evidence that would support a Batesian mimicry system. The greater abundance of the fall brood of *N. terlooii* that coincides with the monarch migration may have facilitated selection for the orange-patterned females. Understanding the evolution of mimicry in *Neophasia* may help us to better understand the biogeography of the monarch butterfly, which has received increased conservation attention in recent years.
